# Risk Perception, Communication and Behaviour Towards Food Safety Issues

**DOI:** 10.3390/foods14020322

**Published:** 2025-01-19

**Authors:** Diogo Thimoteo da Cunha

**Affiliations:** Universidade Estadual de Campinas, Faculdade de Ciências Aplicadas, Laboratório Multidisciplinar em Alimentos e Saúde., Pedro Zaccaria, Limeira 1300, Brazil; dtcunha@unicamp.br

## 1. Introduction

*“In spite of over 100 years of research and millions of dollars spent, food safety remains a worldwide public health issue* (Christoper Griffith cited in Yiannas (2009) [1])”. This emblematic statement continues to reflect the current challenges posed by food safety issues. It is estimated that unsafe food costs low- and middle-income countries around 110 billion USD a year [2]. Developed countries also face a similar challenge. Pathogens such as *Salmonella* spp., *Escherichia coli* and *Listeria monocytogenes* continue to jeopardise societal health, and pose a significant risk to food safety in the United States and Europe [3,4,5]. In this sense, it is reasonable to ask the question why do foodborne illnesses still exist in a connected world despite technological advancements, including the advent of artificial intelligence and various technological devices? There is no direct answer to this question, but many authors in the field would agree that this is because food, at least the majority of it, is farmed, produced, handled and distributed by humans. This ongoing challenge underscores the critical role of the human factor in the entire food production and consumption chain. We do not yet have a one-size-fits-all solution for food safety. But there are some interesting approaches that focus on human behaviour which are promising, even if there are still some obstacles to overcome.

In recent years, research on organisational factors affecting food safety, particularly in relation to food safety culture, has increased significantly [6]. Food safety culture is a deeply rooted organisational construct that encompasses shared beliefs, behaviours and assumptions among all employees within an organisation [7]. The theoretical framework of food safety culture emphasises the importance of effective communication, active leadership and adequate risk perception in promoting a strong food safety culture within an organisation. However, understanding risk perception is a complex endeavour, even within the specific field of food safety research [8]. Risk perception is the judgement of an individual when they are asked to characterise and evaluate an action, practice or technology that is considered hazardous [9]. Experts typically define risk in terms of mortality rates; however, the public perception of risk is influenced by a broader range of factors that go beyond mere danger [10,11]. These factors include perceptions of fairness, control, familiarity and moral implications. Consequently, the risks that pose the greatest threat to human life are not necessarily perceived as the most frightening by the public (see, for example, the COVID-19 pandemic). Furthermore, risk assessments based on technical or scientific data do not always match public perception [11]. This discrepancy often results from the “affect heuristic”, a cognitive bias in which people rely heavily on emotions to guide their judgements and decisions [12]. Consequently, effectively influencing public risk perception presents a significant challenge for us as specialists.

In light of these factors, it is clear that risk is characterised by a series of deliberate decisions made by people who want to achieve the best possible outcome for themselves, or others, given the resources and circumstances available to them [13]. Often, the most desirable outcome takes precedence over efficiency, practicality or feasibility over purely health-related or supportive considerations. Consequently, examining how risk perception, communication strategies and various cognitive variables influence food safety behaviour is both timely and important. The articles presented in this Special Issue contribute significantly to our understanding of how to effectively communicate with the public to improve food safety.

## 2. Overview of Published Papers

Seven articles have been published in this Special Issue (Contributions 1 to 7). Notably, these papers cover a wide range of subjects and approaches to food safety. The authors also examined the perspectives of different stakeholders, including consumers, farmers, food handlers and managers. This section outlines the objective of each contribution and provides a brief discussion of their novel findings, methodological approaches, and identified limitations.

Three articles in this Special Issue focused on food safety among consumers. As the final link in the food safety chain, consumers play a crucial role. Numerous factors, including knowledge, attitudes, risk perceptions, subjective norms and others, have been found to influence consumers’ behaviour, consequently influencing their food safety practises and ultimately their health [14]. Given the important role of meat and meat products in the modern diet and the associated risk of foodborne illness, two studies in this Special Issue have focussed on examining consumer perceptions of meat from different perspectives. Ballout et al. (2024) (Contribution 1) analysed consumer perceptions of raw meat consumption using the knowledge–attitude–practise (KAP) model. Although the KAP model is often criticised [15], it remains one of the most widely used theoretical frameworks for understanding food safety behaviours. Given the limited research on raw meat consumption, the model provides an appropriate starting point for this study. Expanding upon previous research in the field, the authors emphasise the significant influence of attitudes towards and knowledge of food safety on the food safety practises of Lebanese consumers.

Conversely, the work of Charlesworth and Mullan (2024) (Contribution 2) is an example of how the oversimplified KAP model can be improved. In their research, they examined food the safety knowledge, behaviours and associated psychological constructs in individuals at a higher risk of food poisoning and compared the results with the general population. They employed an indirect method to assess risk perception [16]. In this approach, firstly, the participants’ self-perceived risk of experiencing a foodborne illness is measured. The participants are then asked to assess the risk that others in the same situation would have. Participants at a higher risk of food poisoning had higher risk perception scores regarding different food safety behaviours. However, people who have experienced salmonellae infections may have a low perception of risk and an optimistic bias compared to people without such experience [17]. This may be explained by affect heuristics (or risk as feelings), where risk judgments are based on immediate and intuitive responses to adverse events and dangers [12]. By relying on affective impressions rather than tedious calculations and utility maximisation, individuals can make decisions more quickly and efficiently, a strategy that proves sufficient in many situations [18].

As a conclusion to the consumer studies section, Parrella et al. (2024) (Contribution 3) investigated how different perceptions affect US consumers’ behavioural intentions towards irradiated ground beef. As predicted by the authors, objective knowledge, perceived benefits and attitudes were positive predictors of behavioural intention. However, neophobia towards food technology and risk perception were negative factors affecting attitudes and behaviours. It is well known that the lower the perceived benefit, the higher the perceived risk, and vice versa [19]. Their results are consistent with the theory of cognitive consistency. The theory states that people have a strong desire to harmonise their beliefs and attitudes. If people perceive an activity or technology negatively, this can lead to a systematic devaluation of the perceived benefits and an exaggeration of the perceived risks [16]. This tendency stems from a desire to maintain cognitive consistency and ensure that their beliefs and attitudes are aligned. In this sense, the authors emphasise the importance of improving perceived benefits and reducing perceived risk in encouraging the use of this technology.

Another three articles dealt with food safety practises and behaviours. In these studies, different methods were used to assess the risk of foodborne illness or to evaluate compliance with food safety regulations. The significant diversity within the food industry poses a major challenge to comprehensive food safety assessment and management. Canuto et al. (Contribution 4) used a risk assessment approach, focussing on the risk of foodborne illness in Brazilian schools. Using a specialised risk assessment tool, their study showed significant associations between a higher risk of foodborne illness and various socioeconomic factors. These included a lower Human Development Index (HDI), a lower Gross Domestic Product (GDP) per capita, a higher Gini coefficient and a higher Social Vulnerability Index. The finding that schools serving students from lower socioeconomic backgrounds have a higher risk of foodborne illness raises a crucial question: Is access to safe food a privilege reserved for the wealthy? This finding emphasises the importance of ensuring that all people, regardless of their socio-economic status, have access to the human right to adequate food (which includes food safety). In addition, this paper highlights the use of risk assessment tools. Such tools promote good practises and allow the researcher to delve into factors that are actually associated with foodborne illness [20].

Na et al. (2024) (Contribution 5) explored food safety in shared kitchens using different methodological approaches. Shared kitchens are spaces equipped with communal kitchen facilities and are shared by several users. The aim of this study was to assess the microbial and chemical hazards posed by the food prepared and the environment within shared kitchen facilities. The study also included surveying shared kitchen operators and a comparison of regulations between Korea and other countries. This is a timely and novel study as research in this area is limited despite the rapid growth of delivery-only restaurants and dark kitchens [21]. Interestingly, the researchers found that open and shared communal kitchens achieved reasonable results and performed better than segregated individual kitchens. The authors suggest a revision of the current regulations and recognise that strict compliance may be a challenge for some operators of community kitchens. However, these regulations must be strict enough, because food safety is a non-negotiable consumer right.

In another Brazilian study, Rodrigues et al. (2024) (Contribution 6) aimed to develop an educational gamification strategy to enhance the food safety practices of family farmers in public food markets in a city in Northeastern Brazil. This is a very novel approach to enhancing food safety. Gamification refers to the application of game design principles and mechanics to non-game contexts, leveraging the psychological motivations that drive engagement in games to improve participation in real-world activities [22]. The authors observed several improvements following the implementation of the educational strategy. These results emphasise the importance of researching and implementing innovative educational strategies to improve food safety. Recent studies have shown the potential of gamification strategies, but there is still room for further research to truly understand their effectiveness, costs and benefits [23,24].

We closed the Special Issue with a timely review. Berglund, Simsek and Feng (2024) (Contribution 7) conducted a systematic review and meta-analysis that included a thematic synthesis to assess the effectiveness of online food education programmes in improving the knowledge, attitudes and practises of consumers, food workers and students. Their analysis also examined identified barriers and recommendations for improvement. Online approaches offer significant advantages, including cost effectiveness and the ability to reach different target groups. But is this enough to shape food safety behaviour? There is still a long way to go regarding online approaches. The researchers found that students, food workers and consumers had difficulty concentrating because they lacked the time to complete the tasks and had difficulty understanding the material. The knowledge effect size was moderate, suggesting that online food safety education is effective in communicating food safety information. However, knowledge does not always translate into attitudes and practices [15].

## 3. Conclusion and Future Studies

In this Special Issue, readers will find papers that deal with the fundamentals of food safety practices and examine how knowledge, attitudes and risk perception shape behaviour and intentions. This is a valuable collection of papers that utilise sound scientific methods and include relevant references on risk perception and food safety behaviour.

Risk perception is significantly influenced by personal emotions and situations. Future research should investigate further strategies to improve risk perception, even in positive scenarios where consumers and workers may become complacent despite the utilisation of appropriate environmental and safety practises. This is consistent with Slovic’s et al. [12] concept of “risk as analysis”, where risk judgements are primarily determined by logical thinking and careful consideration.

Soft skills training (e.g., communication, job crafting, employee support, flexibility) and the promotion of a proactive food safety culture within organisations hold great promise for improving food safety in the retail, service and industrial sectors. On the consumer side, educational messages need to be communicated effectively to improve risk perception based on credible scientific concepts. It is crucial that we constantly explore new approaches to improve food safety in all sectors, while recognising that human behaviour is influenced by a complex interplay of past experiences, emotions, knowledge, attitudes and various psychological factors which are of paramount importance. If we neglect these factors, we undermine our ongoing efforts to improve food safety.

## Data Availability

No new data were created for this study.

## Abbreviations

The following abbreviations are used in this manuscript:

KAP	Knowledge Attitudes and Practices
HDI	Human Development Index
GDI	Gross Domestic Product
